# Pesticide pollution in India: Environmental and health risks, and policy challenges

**DOI:** 10.1016/j.toxrep.2024.101801

**Published:** 2024-11-09

**Authors:** Urvashi Kashyap, Shivani Garg, Pooja Arora

**Affiliations:** Institute of Environmental Studies, Kurukshetra University, Kurukshetra, Haryana 136119, India

**Keywords:** Pesticides, Water, Soil, Environment, Health

## Abstract

Intensive agriculture practices in India to meet the food demand of the increasing population have led to the use of agrochemicals such as pesticides in higher quantities to increase productivity resulting in contamination of the environment. Pesticides control pests, weeds, and diseases in plants, animals, and humans. Despite bans on pesticides such as organochlorides (OC), organophosphate (OP), or synthetic pyrethroids ranging from minimal to excessive, are detected in soil, surface water, and groundwater often exceeding WHO and BIS safety limits. The predominantly found pesticides were DDT, HCH, Endosulfan, malathion, chlorpyrifos, atrazine, endrin, cypermethrin, dichlorvos, etc. Different ranges of pesticides were detected in different states (Kashmir, UP, Tamil Nadu, Kerala, Rajasthan, Haryana, Assam, Madhya Pradesh, etc.) of India, which demonstrate that pesticides can persist in the environment and later can show bioaccumulation in the food chain. The article explores the consequences of this pollution such as biomagnification, bioaccumulation, and risks to human health and ecological integrity. This article also covers the adverse effects of pesticides such as carcinogenic, teratogenic, mutagenic, and endocrine-disrupting properties along with the importance of developing new policies or strengthening the current policies and regulations to monitor the use of pesticides.

## Highlights

• Intensive agriculture in India has resulted in indiscriminate use of pesticides to meet growing food demand.
• Various pesticide residues have been detected in surface water, groundwater, and soil above WHO and BIS limits.
• Need for new or strengthened policies to monitor and regulate pesticide use.
• Innovative techniques required to remove pesticides from the environment.
• Emphasis should be on raising awareness to address pesticide contamination in India.

## Graphical Abstract

**Figure:**
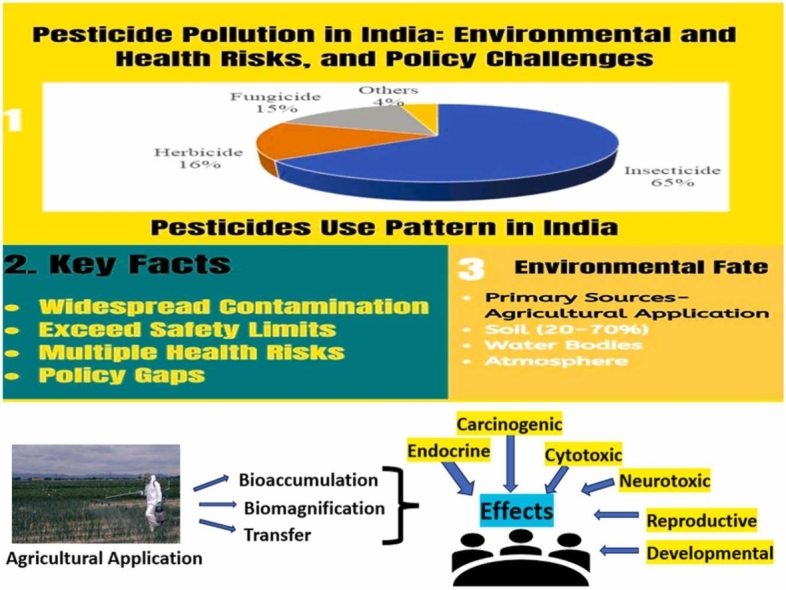

## 1 Introduction

**Fig. 1:**
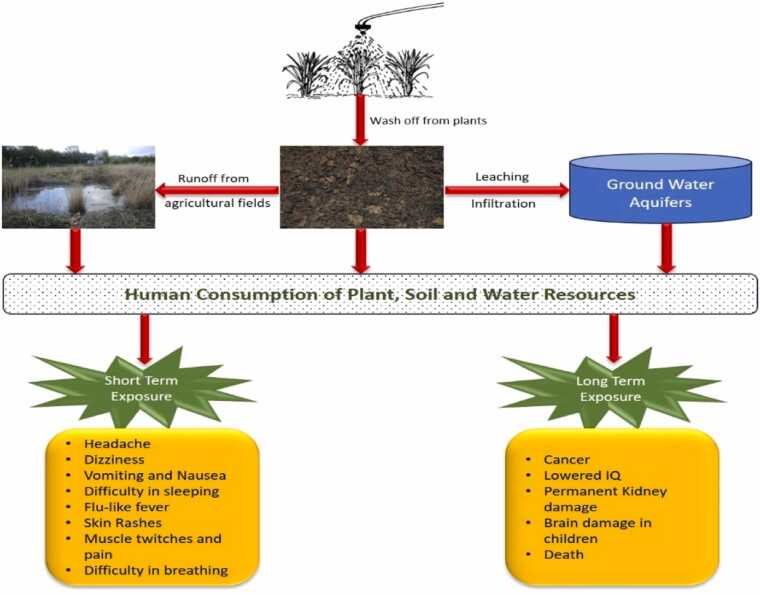
Health issues associated with pesticide exposure.

The agriculture sector plays a pivotal role in sustaining human existence, while the ever-increasing global population is putting tremendous pressure on the food supply based on the practice [117]. Out of various rigorous procedures associated with agriculture, a noteworthy one is the application of chemicals to manage pests and unwanted plants and improve food yield [111], [37], [7]. They are of vital importance in agriculture to safeguard the crops but their application can have notable repercussions on human health and the environment [5]. Chemicals known as pesticides are employed to eradicate a wide range of pests that pose a threat to crops, livestock, and overall farm productivity [109]. Furthermore, they have versatile applications, serving as plant growth regulators (to either stimulate or hinder growth), defoliants (inducing leaf or foliage shedding), desiccants (speeding up plant tissue drying artificially), and nitrogen stabilizers (inhibiting nitrification, denitrification, ammonia volatilization, or urease production by affecting soil bacteria) [116], [41]. They include more specific categories of herbicides, insecticides, weedicides, rodenticides, fungicides, piscicides, etc. KANKAM [67]. Pesticide contamination accounts for approximately 25 % of global soil degradation and contributes to 30 % of biodiversity loss in agricultural landscapes [81]. Nonetheless, chemical pesticides tend to lack specificity in targeting, potentially leading to harm to unintended species, and many of these substances persist in the environment for longer duration [34]. They not only affect the target organisms but also affect the environment including air, soil, and water (surface or ground) by leaching, flow, percolation, and pulverization process [96], therefore contaminating the whole ecosystem [139]. While extensive research exists on pesticide impacts on individual environmental components, there remains a critical gap in understanding the cumulative and synergistic effects of multiple pesticides across different matrices, particularly in developing nations where monitoring systems are less robust. This review is especially timely given the recent surge in intensive agriculture practices and the emergence of new pesticide formulations, necessitating a comprehensive assessment of their environmental fate and ecological implications. Depending on the plant's characteristics and density, it is estimated that approximately 35–50 % of the pesticide material used settles in the soil right away after the spraying process. These pesticides affect the soil through direct application or via wash-off coming from the plant surface during rains. They also get infiltrated to reach the groundwater and contaminate it. Pesticide endurance, mobility, and metabolic processes determine its interaction with soil and groundwater. The potential water solubility and binding ability of the pesticide are crucial for the creation of pesticide residues in the soil [65]. It has been reported that about three million individuals worldwide are exposed to poisoning from pesticides annually leading to around 200,000 fatalities, primarily concentrated in developing nations [154], [46]. These numbers might be even higher due to potential underreporting and the absence of credible data. These pesticides show a broad spectrum of immediate and prolonged health effects which include neurological damage, reproductive effects, cancer, birth defects, immune suppression, etc. [26], [151], [143]. Short-term and long-term exposure to pesticides may cause several health issues (Fig. 1). The present study is intended to provide a comprehensive decadal evaluation of various pesticide residues in ground water, surface water, soil and their impacts on human health and biodiversity with special reference to India.

## 2 Methodology

A systematic literature search to identify relevant studies on pesticide pollution in Indian agroecosystems focused on water and soil contamination was conducted. The databases used are Scopus, Web of Science, Google Scholar and PubMed. Search terms included the keywords and combination of keywords: pesticides, pollution, contamination, India, agroecosystems, soil health, water, residues, organophosphates, synthetic pyrethroid, etc. The search was limited to studies published between 2012 and 2023 to ensure the inclusion of recent data and trends. Inclusion criteria include studies conducted in India, peer-reviewed articles, quantitative data on pesticide residues, focus on pesticide pollution on soil or water in agricultural ecosystems. The selected articles are characterised and analysed by geological location within India, types of pesticides studied, environmental compartments such as soil, surface and groundwater, sampling methods and frequency and analytical techniques used for pesticide detection and quantification. Potential selection biases were addressed by using multiple databases to ensure comprehensive coverage, and consideration of studies from all regions of India, although publication bias may favour more extensively studied agricultural regions. This systematic approach aimed to provide a comprehensive and unbiased representation of pesticide pollution research in Indian agroecosystems while acknowledging inherent limitations in the selection process.

## 3 Pesticide usage in Indian agriculture

**Fig. 2:**
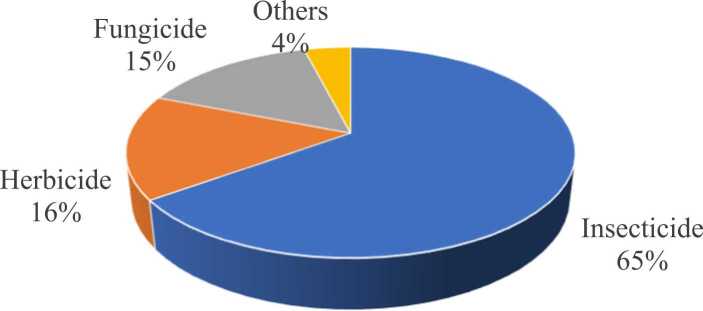
Pesticide use pattern in India.

India, being an agrarian economy with a substantial portion of its population dependent on agriculture, has witnessed a substantial increase in pesticide usage over the past few decades. India holds the fourth position in the production of agrochemicals globally, however, the consumption of pesticides is about 0.5 kg per hectare which is significantly lower than in other nations such as Japan (12 kg per hectare), Korea (6 kg per hectare), and USA (4.5 Kg per hectare). Among the worldwide pesticide production of 2 million tons annually, India's consumption stands at a mere 3.57 %. In India, pesticides are applied to only 25 % of the total cultivated land (Fig. 2), which covers 16.7 million hectares, but this limited usage has had significant environmental repercussions [40]. The country has approximately 330 insecticide/ pesticide molecules registered for usage (CIB & RC, [27]) out of which the usage of insecticides accounts for the highest consumption (65 %) followed by herbicides and fungicides.

**Fig. 3:**
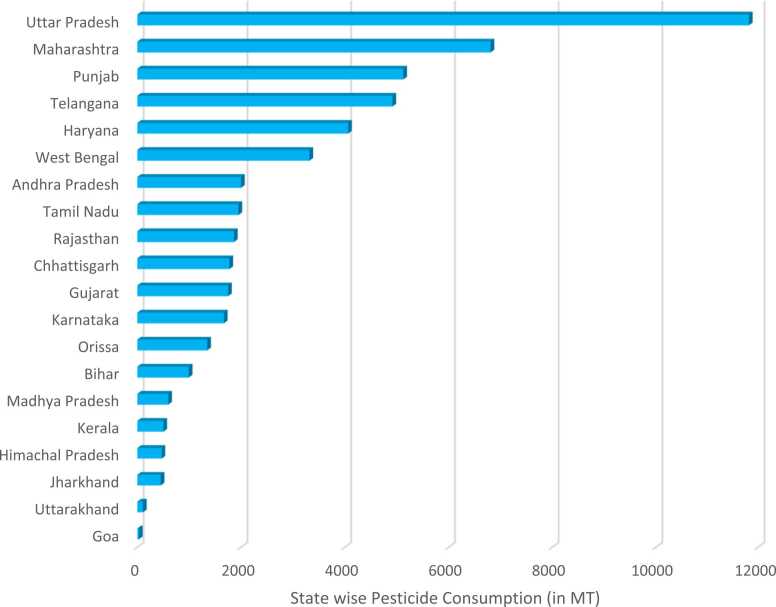
State-wise pesticide consumption in India in 2022–2023.

The intensive application of pesticides is prevalent in almost every agricultural state of India. As per the statistical data from the Ministry of Agriculture & Farmers Welfare [82], Uttar Pradesh accounted for the highest consumption of chemical pesticides in 2022–23 followed by Maharashtra and Punjab. The last consumption was reported in the state of Goa (Fig. 3). Further, this usage is determined by the variety of crops being grown, pest prevalence, climatic conditions, and the attraction towards growing high-yielding varieties. However, the overreliance on these harmful agrochemicals has raised concerns about their potential impacts on the environment and human health.

The use of pesticides has increased in some parts of India for several interconnected factors. Agricultural intensification has been a primary reason across many regions, aimed at increasing food production to meet a growing population. Increased pest pressures have, in turn, resulted from the altered distributions of pests and crop vulnerabilities under climate change (unpredictable weather like increase in temperature and decrease in rainfall), particularly in states such as Punjab and Haryana [125]. Crop diversification efforts, especially concerning high-value cash crops and vegetables in Maharashtra and Gujarat, have introduced more intensive crops for pest management. This is especially so in cotton-growing areas where pesticide use remains high despite the introduction of Bt cotton [16]. Socio-economic factors remain highly important, with many small and marginal farmers lacking the knowledge or resources to adopt alternate pest control methods such as Integrated Pest Management (IPM). These are particularly common in states with lower agricultural extension service coverage, such as parts of Uttar Pradesh and Bihar. In some of the states, policies and subsidy structures inadvertently increased chemical pesticide usage. Punjab is one such agricultural policy that gradually promoted input-intensive farming practices over time [102]. Additionally, the increased application of pesticides for potential pesticide resistance development has resulted in farmers opting for more frequent application or higher strength formulation, a new trend noticed in the intensive agricultural belts across India [129]. This has fuelled the rising pesticide usage in regions like parts of Maharashtra and Karnataka [2]. The situation also becomes murkier as there has been irregular implementation of pesticide usage regulations in the states.

The rise in pesticide use in Uttar Pradesh is attributed to several factors. The increase in food demands resulted in agricultural intensification, thus contributing to the pressure of pest [74]. Climate change (change in weather pattern like warmer temperature, decreased rainfall) resulted in a shift in the distribution of the pest as well as crop susceptibility. During the dry season when temperature is high, pest activities are increased and the climate of Uttar Pradesh makes it favourable for the pest to grow. Therefore, pesticide use increased with frequent applications [105]. Crop diversification efforts in cash crops (cotton, sugarcane) and vegetables by the state, introduced crops that often require high-level pest management. The reliance on pesticides as a quick-fix solution has become more pronounced [115]. Economy constraints and lack of awareness of alternatives, such as Integrated Pest Management (IPM), amongst small and marginal farmers means dependency on chemical pesticides continues. Government policies and subsidy structures favoring chemical inputs encouraged their use over more sustainable practices [74]. Additionally, the development of pest resistance due to chronic use of pesticides has made the farmers apply those substances at higher intensities or to a more potent formulation. The commercial pressures from pesticide companies in addition to the changes in the landscape of water availability (depletion in groundwater) and the effects thereof on crop health also contribute to the situation at hand [115], [3].

In India, farmers are also shifting towards the use of biopesticides and organic farming after seeing the effect of synthetic pesticides. Biopesticides are pesticides that are naturally produced from living organisms such as bacteria, plants, herbs etc. Chandler et al., [32]. They are less harmful to living systems, so are safer to use than synthetic pesticides. For the production of decent yield of crops, pesticide application is necessary to stop the damage caused by the infestation of pests in the agricultural fields. Biopesticides are a better option to adopt as man-made pesticides are expensive and pests become resistant to the continuous use of these pesticides and make them ineffective. Examples of biopesticides are *Bacillus thuringiensis* (microorganisms), *Azadirachta indica* (botanical products), *Beauveria bassiana* (fungal species) [118], [23], [33], [68]. Biopesticides are biodegradable and efficiently remove the pest, also for plants, they increase the nutrient availability in soil [11], [50]. Therefore, it forms a crucial part of integrated pest management practices [31]. The consumption of biopesticides in India constitutes about 9 % of the total pesticides consumed [158] and, as estimated for the year 2050, will reach up to 50 % of the overall pesticide market [72]. The annual growth rate is predicted to be 2.5 per cent [158]. However, till now, the market for biopesticides still fails to develop according to its projections. It is also almost low in comparison with synthetic pesticides in the market [72].

Organic farming is a production system that avoids or largely excludes using synthetically compounded fertilizers, pesticides, growth regulators, and livestock feed additives. It relies on crop rotations, crop residues, animal manures, legumes, green manures, off-farm organic wastes, and aspects of biological pest control to maintain soil productivity and tilth, supply plant nutrients, and control insects, weeds, and other pests [92]. Organic farming helps in the improvement of soil health, water conservation, and biodiversity enhancement as well as farmer's economic benefits. In 2016, Sikkim became India's first fully organic state. The government intended to make Sikkim an organic state in 2003 and started by converting farmland into organic farming by 2016 [57]. While other states making progress in organic farming such as Uttrakhand, Kerala, Mizoram, Meghalaya, Karnataka, Madhya Pradesh etc.

## 4 Classification of pesticides

Pesticides can be classified into three categories depending on their major chemical constituents i.e, 1. Organochlorines 2. Organophosphates and 3. Carbamates. The important properties of these are summarized in Table 1.

**Table 1:** Important properties of pesticides.

**Property**	**Organochlorine**	**Organophosphate**	**Carbamates**
Structure			
Functional group	It contains carbon, hydrogen, and chlorine as functional groups.	It contains phosphorus as a functional group.	These are essentially esters of carbamic acid. It contains a carbamate functional group.
Persistence	These are persistent in the environment and break down slowly.	These are not persistent in the environment.	These are not persistent in the environment.
Bioaccumulation	These organic compounds show bioaccumulation	These compounds are not bioaccumulative.	These compounds are not bioaccumulative.
Biomagnification	These compounds biomagnify through the food chain	They do not show biomagnification	They do not show biomagnification.
Health effects	Many acute and chronic disorders are caused by OC. Acute poisoning symptoms are dermal irritation, tremors, dizziness, headache, respiratory problems, nausea, and seizures. Chronic diseases include cancer, birth defects, neurological damage, abnormal immune system function, respiratory illness, and Parkinson’s disease.	OP inhibits the synthesis of an enzyme known as cholinesterase (ChE). Signs of exposure encompass queasiness, migraines, muscle twitches, shivering, profuse salivation and teary eyes, breathing difficulties due to diaphragm paralysis, seizures, and at elevated doses, fatality.	The acetylcholinesterase enzyme is temporarily deactivated by carbamates, leading to ChE inhibition poisoning. Symptoms include vomiting, diarrhea, exhaustion, convulsions, difficulty in breathing, etc.
Examples	DDT, BHC, and its metabolites (α, β, γ) methoxychlor, Endosulfan endrin, and aldrin.	Malathion, Parathion, phorate, chlorpyrifos, and fenthion.	Aldicarb, fenoxycarb, furadan, carbaryl (Sevin), and carbofuran

Among all the pesticides, Organochlorines (OC), organophosphates (OP), carbamates, and synthetic pyrethroids (SP); organochlorine pesticides (OCP) have contributed to 40 % in the Indian scenario. They are highly bio-accumulative and thereby show biomagnification in the food chain and later in the food web. The biological toxicity thus caused by these substances is of great concern [140]. Some of the prominent examples of such pesticides are dichlorodiphenyltrichloroethane (DDT) and its derivatives; dichlorodiphenyldichloroethane (DDD); dichlorodiphenyldichloroethylene (DDE); aldrin; dieldrin; polychlorinated biphenyl (PCB), hexachlorocyclohexane (HCH) etc.

Though in the last decade, organophosphate pesticides (OPP) have dominated over the use of OCP, also the use of carbamates and SP has increased.

The initial gathering of the Stockholm Convention in 2001 laid down the framework for eliminating or regulating the use of substances showing traits like persistence, bioaccumulation, toxicity, and long-distance environmental transportation, thereby defining Persistent Organic Pollutants (POPs). While only a dozen pesticides have been categorized as such, they serve as prominent illustrations of POPs [119]. POPs are manufactured toxic substances with extended long-term endurance in the environment even at low concentrations. When these substances come in contact with the organisms, they are ingested via inhalation, oral, or dermal route, and can accumulate in the trophic chain. Also, they are responsible for causing severe animal and human health problems [135], [52], [93].

Pesticides can also be classified according to the target organisms they are designed to control or act at (Table 2) and also according to their hazard potential (Table 3)

**Table 2:** Pesticide classification according to their target organisms.

**S. No.**	**Pesticide**	**Target Organism**	**Examples**
1.	Insecticides	Insect	DDT, Benzene hexachloride (BHC)
2.	Herbicides	Weeds	Borax, Nitrofen
3.	Rodenticides	Rodents	Warfarin, zinc phosphate
4.	Fungicides	Fungi	Bordeaux mixture
5.	Algaecides	Algae	Copper sulfate, Endothal
6.	Bactericides	Bacteria	Dichlorophen, Oxolinic acid
7.	Nematicides	Nematodes	Phorate, 1,2-dibromo−3-chloropropane (DBCP)
8.	Avicides	Birds	Strychnine, Avitrol
9.	Miticides	Mites	Azobenzene, Dicofol
10.	Molluscicides	Snails	Sodium pentachlorophenol
11.	Piscicides	Fishes	Trifluoro methyl nitrophenol (TFM)

**Table 3:** Pesticide classification is based on hazard potential [155].

**Class and Category**	**LD50 for the rat (mg/kg body weight)**		**Some examples of active ingredients in pesticides**
	**Oral**	**Dermal**	
**Class Ia** **Extremely hazardous**	<5	<50	Aldicarb, Brodifacoum, Flocoumafen, Sodium fluoroacetate, hexachlorobenzene, Phorate, Mercuric chloride etc.
**Class Ib** **Highly hazardous**	5–50	50–200	Sodium Cyanide, Carbofuran, Thiofnox, Coumaphos, Sodium arsenite, Warfarin, cyfluthrin, beta-cyfluthrin, triazophos, dichlorvos, dicrotophos, fenamiphos, methiocarb, monocrotophos, etc.
**Class II** **Moderately hazardous**	50–2000	200–2000	Azocyclotin, Bendiocarb, Benzovindiflupyr, Endosulfan, Endothal-sodium, Esfenvalerate, Fipronil, Gamma-HCH, Lindane, Methyl iodide, Methyl isothiocyanate, Proppoxur, Quinalphos, Thiodicarb, Triazamate etc.
**Class III** **Slightly hazardous**	> 2000	>2000	Acetochlor, Alloxydim, Ametoctradin, Anilazine, Benfuresate, Bispyribac, Buprofezin, Chinomethionat, Chloridazon, Cyclaniliprole, Cyflumetofen, Diafenthiuron, Dichlormid, Diflufencan, Dimethirimol, Dinotefuran, Fenarimol, Fosamine, Hexaconazole, Metazachlor, Penconzole, Quinclorac, Undecan−2-one etc.
**Class U** **Unlikely to present acute hazard**	5000 or higher		Amitrole, Benodanil, Bromacil, Captan, Chlorfluazuraon, Cinosulfuron, Dalapon, Daminozide, Ethirimol, Fosetyl, Maneb, Napropamide, Picloram etc.

The diversity of pesticide classes reflects the varied challenges faced in crop protection and the need for targeted solutions.

## 5 Environmental fate of the pesticides

**Fig. 4:**
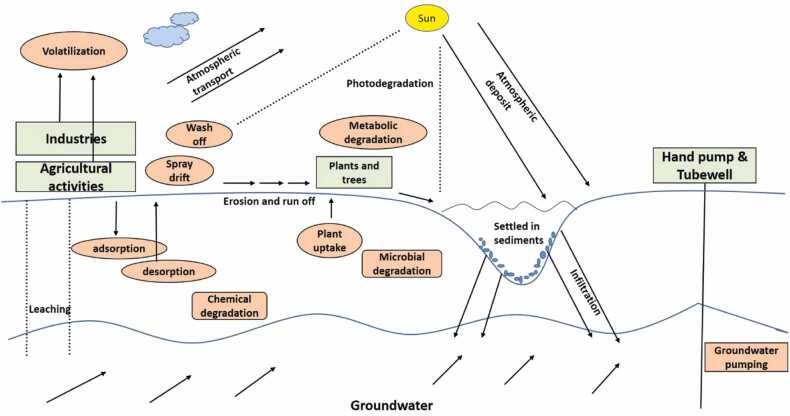
Pesticide fate and transport in the hydrogeological system.

The Presence of pesticides is everywhere, in water (surface and ground), in soil, sediments, air, and in organisms’ tissue like fish, birds, plants, and humans. Their introduction, transport, and fate in different environmental components such as soil, water, and air are determined by various factors. Pesticides end up in different parts of the atmosphere or the organisms. Through water, pesticides can end up in the sediments, and aquatic organisms, which show bioaccumulation and later biomagnify in the trophic level. Whereas through the soil, pesticides can enter the surface or groundwater and even the plants or crops take up the pesticide residues which are ultimately received by the different organisms and humans that come in contact with these things and show toxicity and harm to the population (Fig. 4).

Soil serves as the primary repository for pesticides that are applied in agriculture. About 10 % of the pesticides reach the target organisms whereas approximately 20–70 % of the used pesticides and their metabolites reach the soil and remain there as persistent residues [106].

Fumigants, herbicides, and nematicides pesticides are applied directly to the soil for plant disease and pest control [121]. However, once a molecule of pesticide reaches the soil, its fate in the environment is determined by the soil characteristics, pesticide properties, and climatic conditions [152]. The introduction of pesticides into the hydrogeo- environment (ground and surface water, soil, and sediments) includes sewage discharge, urban runoff, runoff induced from deforestation, discharge from domestic and industries, etc. [18], [58]. The fate of the pesticide’s residues (including degradation, retention, and mobility) in the soil is determined by the biological and physico-chemical properties which include type of soil, soil texture, pH, moisture content in soil, temperature, organic matter, clay content, mineral composition (Arias-Estevez et al., 2008; [150]). The entry of pesticides into the surface water is through wastewater discharge, runoff, spills, and atmospheric deposition [29]. They can reach and contaminate the groundwater by leaching from the agriculture fields, washing sites, mixing sites, and waste disposal sites [103].

The fate and mobilization of pesticides in water environment or aquatic bodies are determined by the physicochemical properties which include chemical nature, volatility, aqueous solubility, octanol-water partition, concentration, adsorption coefficient, half-life, chemical composition, degradation potential, etc. Arias-Estévez et al., [61], [8], [86].

Pesticides can be distributed and can contaminate the atmosphere globally and it has a significant pathway of transport [18], [107], [108], [120], [58]. The entry of pesticides into the atmosphere can be either by wind erosion of soil that is treated with pesticide, drift application, or vaporization of pesticide after post-application. The pesticides and their degraded products may be transported long distances before they return to the earth’s surface by the removal process of wet and dry deposition of the atmosphere [28], [29].

## 6 Pesticide contamination in Indian context

The soil and water contamination caused by pesticides is a critical environmental issue in India with long-term and far-reaching consequences for ecosystems and human well-being. The residues of pesticides have the potential to persist in the environment for extended periods, accumulate in the food chain, and thereby have adverse impacts on soil fertility, microbial activity, and biodiversity. Furthermore, these pesticides get their entry into the water bodies (rivers, lakes, groundwater aquifers) via leaching and runoff and pose deleterious effects on aquatic life and human health due to the consumption of contaminated water.

Pesticides have been extensively used in India to increase crop productivity and used as healthcare for humans and animals. Pesticide consumption in India is around 0.5 kg per ha, which is very low compared to other developed countries and that is attributed to multiple factors such as predominantly small-scale farming practices where many farmers lack financial resources for extensive chemical inputs, the significant adoption of traditional agricultural methods in many regions, and varied state-level policies promoting organic farming and IPM [110]. Approximately 330 insecticide/pesticide molecules that have been registered in India for usage [83] are used in higher quantities and have a significant impact on the environment. The pesticides have a long transport range in the atmosphere in India due to the diverse distribution of climate that ranges from temperate in the Himalayas, tropical in the south, and subtropical in the north [30]. The present study reports various pesticides from different states in the soil and water of India. Different methods have been used for the analyses of the pesticides. The study showed that there were varying degrees of pesticide contamination in the soil and water (Table 4)

**Table 4:** Pesticide contamination in different states of India.

**Place/state**	**soil/water**	**Pesticide detected**	**Method used**	**Reference**
Ambala & Gurgaon Hisar	Groundwater Surface water	∑HCH- 7.1–298.6 ng/l & 8.4–288.6 ng/l ∑DDT- 89.3–1888.4 ng/l & 182.1–665.1 ng/l ∑Endosulfan- BDL- 164.2 ng/l & BDL- 913.8 ng/l ∑HCH- 2.3–560.6 ng/l ∑DDT- 50.1–332.2 ng/l ∑Endosulfan- BDL−206.3 ng/l	GC-ECD	Kaushik et al., [71]
Kerala (Kasaragod district)	Groundwater (open wells)	α-BHC- 8.8 μg/l β-BHC- 2.3 μg/l γ-BHC- 33 μg/l α-endosulfan- 58 μg/l β-endosulfan- 0.05 μg/l	GC-ECD	Akhil, Sujatha [4]
Assam Dibrugarh district Nagaon district	Soil Soil	∑HCH- 178–1701 ng/g ∑DDT- 75–2296 ng/g ∑HCH- 98–1945 ng/g ∑DDT- 166–2288 ng/g	GC-ECD	Mishra et al., [86]
Karnataka (Chamarajanagar district)	Soil Rice field water Subcanal water River water	∑HCH−19.8 μg/kg ∑Endosulfan−68.2 μg/kg ∑DDT−52 μg/kg α-Cypermethrin- 8.6 μg/kg Fenvalerate−19.2 μg/kg ∑HCH−7.55 µg/l ∑Endosulfan−2.575 µg/l ∑DDT−5.075 µg/l α-Cypermethrin−0.925 μg/l Fenvalerate- 0.475 μg/l ∑HCH−2.15 μg/l ∑Endosulfan−32.265 μg/l ∑DDT- 41.25 μg/l α-Cypermethrin−0.2475 μg/l Fenvalerate−20.575 μg/l ∑HCH−2.3 μg/l ∑Endosulfan−15.4 μg/l ∑DDT−3.6 μg/l α-Cypermethrin−0.5 µg/l Fenvalerate−12 µg/l	GC-ECD	Mahdavian, Somashekar [78]
Tamil Nadu (North zone- Coimbatore, Erode, Pollachi, Bhavani)	Water (surface irrigation water)	Dichlorvos- 4.421 µg/ml Chlorpyrifos- 1.329 µg/ml	GC-MS	Sukirtha, Usharani [138]
Northeastern part (Assam, Tripura, Manipur)	Soil	ΣDDT- 49.6–5068 pg/g ΣHCH- 9.5–2850 pg/g ΣEndosulfan−26.9–1990 pg/g	GC-ECD	Devi et al., [39]
Godavari delta	Surface water	Endosulfan- 0.03–0.84 μg/l Chlorpyrifos- 10.54–30.25 μg/l Monocrotophos- 10.25–25.5 μg/l α-HCH- 0.03–0.145 μg/l β-HCH- 0.02–0.095 μg/l γ-HCH- 0.27–0.69 μg/l 2,4 DDE- 0–0.455 μg/l 2,4 DDD- 0.21–0.58 μg/l	GC-ECD	Mohammed, Penmethsa [87]
Sonipat Dehradun	Soil Water Soil Water	Imidacloprid- 0.05 mg/kg Chlorpyrifos- 0.03–0.05 mg/kg Hexaconazole- 0.01 mg/kg Carbendazim- 0.001–0.05 mg/kg Tricyclazole- 0.002 mg/kg Propiconazole- 0.5 mg/kg Lambda-cyhalothrin- 0.05 mg/kg No pesticide was detected in any water sample Tricyclazole- 0.003 mg/kg No pesticide was detected in any water sample	GC-MS	Arora et al., [10]
Maharashtra (Bhandara, Amravati and Vavatmal) (Bhandara, Amravati and Vavatmal)	Groundwater Surface water	HCH- 0.06, 0.39 and 0.08 μg/l Endosulfan- 0.72, 0.6 and 0.78 μg/l Dichlorvos- 0.09, 0.8 and 0.07 μg/l Chlorpyrifos- 0.25, 0.11 and 0.18 μg/l Parathion-methyl- 0.03, 0.09 and 0.02 μg/l HCH- 0.06, 0.39 μg/l and N.D Endosulfan- 0.08, 0.42 μg/l and N.D Dichlorvos- 0.20, 0.20 and 0.25 μg/l Chlorpyrifos- 0.44, 0.26 and 0.44 μg/l Phorate- 0.31, 0.19 and 0.33 μg/l Parathion-methyl- 0.42, 0.15 and 0.17 μg/l	GC-ECD and GC-MS	Lari et al., [75]
Kerala (Idukki district)	Soil	p,p’-DDT- 0.018–0.07 mg/kg p,p’-DDD−0.012 mg/kg p,p’-DDE−0.015–0.09 mg/kg Endosulfan sulfate- 0.014–4.00 mg/kg α-endosulfan−0.13–0.33 mg/kg β-endosulfan- 0.03–1.14 mg/kg Ethion- 0.039–0.83 mg/kg Quinalphos- 0.03–0.41 mg/kg Profenophos- 0.025–0.21 mg/kg Chlorpyrifos- 0.025–0.64 mg/kg Lambda-cyhalothrin- 0.09–1.22 mg/kg Bifenthrin- 0.14–1.23 mg/kg Cypermethrin- 0.049 mg/kg Imidacloprid- 0.077–0.08 mg/kg Indoxacarb- 0.154 mg/kg	GC-ECD & FPD and GC-MS	Beevi et al., [14]
Tamil Nadu (Kanyakumari district)	Surface water (agricultural sector)	∑HCH- 0.80–6.99 ng/L ∑DDT- 1.12–7.12 ng/L ∑Endrin (Endrin+Endrin aldehyde+Endrin ketone)- nd–1.84 ng/L ∑cyclodiene (Aldrin+Dieldrin+Heptachlor+Heptachlor epoxide)- 0.62–3.35 ng/L ∑Endosulfan (Endosulfan I+Endosulfan II+Endosulfan sulfate)- nd–5.82 ng/L	GC-MS	Jeyakumar et al., [66]
Punjab	Surface water and groundwater	γ-HCH- 321.9–413.5 ng/l pp-DDT- ND−501.1 ng/l pp-DDE- 579.5–617.7 ng/l Endosulfan sulfate- ND−410.9 ng/l Chlorpyrifos- 1245.4–2808.9 ng/l Profenofos- 322.1–389.9 ng/l Ethion- ND−452.1 ng/l	GC-ECD and GC-MS	Kaur et al., [70]
Maharashtra (Nashik district)	Soil	Carbendazim- 3.68–86.8 µg/kg Azoxystrobin- 1.96–35.2 μg/kg Imidacloprid- 1.47–31.5 μg/kg Flusilazole- 0–2.07 µg/kg Dimethomorph- 1.84–63.7 µg/kg Thiamethoxam- 1.09–24.8 µg/kg Fenamidone- 4.26–13.8 μg/kg Pyraclostrobin- 1.2–23.7 μg/kg Clothianidin- 1.47–7.92 µg/kg Iprovalicarb- 1.13–65.6 µg/kg Hexaconazole- 2.6–4.89 µg/kg Kresoxim methyl- 0–7.69 μg/kg Triadimefon- 0–28.8 μg/kg Penconazole- 0–2.48 μg/kg Spinosad A- 0–2.15 μg/kg pp-DDE- 5.2–5.5 µg/kg pp-DDT- 2.0–2.5 µg/kg pp-DDD- 1.9–3.2 µg/kg	GC-MS/MS and LC-MS/MS	Patil et al., [101]
Madhya Pradesh (Chhindwara district)	Groundwater Surface water	α-HCH- ND−0.16 ppb β-HCH- ND−0.15 ppb δ-HCH- ND−0.086 ppb Endosulfan I- ND−0.29 ppb Endosulfan II- ND−0.16 ppb No pesticide detected	GC-ECD	Bhimte, Meshram [20]
Maharashtra (Parbhani district)	Groundwater	Endosulfan- 0.0017–0.0022 mg/l Heptachlor- 0.0005 mg/l HCH- 0.0056–0.0070 mg/l DDT- 0.0019–0.0042 mg/l Phosphamidon- 0.0010–0.0040 mg/l Dimethoate- 0.1122–0.1543 mg/l Malathion- 0.0067–0.0823 mg/l Chlorpyrifos- 0.0014–0.0412 mg/l	GC-ECD	Motekar Shrinivas [90]
Andhra Pradesh (Prakasam district)	Water (pooled water of paddy fields)	α-HCH- 0.000082 ppm β-HCH- 0.000040 ppm δ-HCH- 0.000067 ppm γ-HCH- 0.000418 ppm Dieldrin- 0.000025 ppm β-Endosulfan−0.000860 ppm p,p-DDD- 0.000062 ppm o,p-DDT- 0.000228 ppm	GC-MS	Tata Rao et al., [141]
Maharashtra (Jalgaon district)	Soil Groundwater	Aldrin- 0.03 ppb 2,4 D- >0.03 ppb BHC- >0.03 ppb Carbonyl- >0.03 ppb Chloropyriphos- >0.03 ppb DDT- >0.03 ppb Endosulfan- >0.03 ppb Ethon- >0.03 ppb Lindane- >0.03 ppb Malathion- >0.03 ppb Aldrin- 0.03 ppb 2,4 D- >0.03 ppb BHC- >0.03 ppb Carbonyl- >0.03 ppb Chloropyriphos- >0.03 ppb DDT- >0.03 ppb Endosulfan- >0.03 ppb Ethon- >0.03 ppb Lindane- >0.03 Malathion- >0.03	GC/MS	Baride et al., [12]
Madhya Pradesh (Gwalior district)	Tighra reservoir (surface water)	HCB- 0–6.3 ng/l α-BHC- 0–1.311 ng/l β-BHC- 0–3.01 ng/l γ-BHC- 0–0.12 ng/l Heptachlor- 0–3.064 ng/l Aldrin- 0–4.2 ng/l α-endosulfan- 0–9.7 ng/l β-endosulfan- 0–12.62 ng/l p,p-DDE- 0–2.42 ng/l Dieldrin- 0–13.4 ng/l o,p-DDD- 0–7.82 ng/l p,p-DDD- 0–11.036 ng/l p,p-DDT- 0–7.9 ng/l Endrin- 0–8.0621 ng/l Chlorpyrifos- 0–12.27 ng/l Methyl parathion- 0–4.21 ng/l Diazion- 0–16.23 ng/l Dichlorvos- 0–22.3 ng/l Ethion- 0–6.85 ng/l Malathion- 0–36.24 ng/l Parathion- 0–6.43 ng/l	GC-MS	Mamta et al., [79]
West Bengal (Deomoni river from Terai region)	River	Chlorpyrifos−0.0091 ppm Dicofol−0.0180 ppm Ethion−0.0892 ppm	HPLC	Singh et al., [128]
Kerala (Idukki district)	soil	ΣEndosulfan- 0.02–0.87 mg/kg ΣDDT- 0.01–0.087 mg/kg ΣOrganophosphorus (chlorpyrifos+Quinalphos)- 0.01–0.589 mg/kg	GC-ECD, GC-FPD and GC-MS	Jacob, Resmi [63]
Tamil Nadu (Pakkam village of Tiruvallur district)	Soil (up to 40 cm)	α-endosulfan- 0.6–4.6 mg/g β-endosulfan- 0.3–3.1 mg/g α- BHC- ND−0.39 mg/g γ-BHC- ND−0.3 mg/g β-cyfluthrin- ND−6.64 mg/g op-DDE- ND−0.009 mg/g Chlordane isomers- ND−0.0006 mg/g	GC-MS	Odukkathil, Vasudevan [98]
Haryana (Sirsa, Panipat, Karnal, Fatehabad, Hisar, Jhajjar, Bhiwani, Rewari, Rohtak, Jind, Kaithal, Yamuna Nagar, Kurukshetra, Ambala, Mewat, Faridabad and Narnaul)	Soil	Phorate- 0.38–0.43 mg/kg Malathion- 0.15–13.89 mg/kg Chlorpyriphos- 0.10–7.12 mg/kg Pendimethalin- 0.06–3.27 mg/kg Butachlor- 0.06–1.69 mg/kg Endosulfan-I- 0.19–047 mg/kg DDT isomers- 0.05–0.95 mg/kg Lambda-cyhalothrin- 0.23–0.25 mg/kg Fipronil- 0.25 mg/kg Atrazine- 0.2–4.52 mg/kg Pretilachlor- 0.05–15.67 mg/kg Propiconazole- 0.14–0.50 mg/kg Triazophos- 0.17 mg/kg	GC-MS	Mishra et al., [84]
Kerala (Kuttanad agroecosystem)	Soil	BHC- 0.01–9.55 ng/g DDT- ND−4.55 ng/g DDE- ND−2.18 ng/g DDD- ND−2.07 ng/g α-endosulfan- 0.74–8.9 ng/g Aldrin- 1.96–2.73 ng/g Dieldrin- 1.29–3.72 ng/g Heptachlor- 1.01–8.52 ng/g	GC-ECD and GC-MS	Sruthi et al., [136]
West Bengal (Kalna Ghat, Rani Nagar Ghat, Iswari Ghat, Halisahar Ghat)	River water Pond Water Tube well water	T-HCH- 0.465–4.132 ng/ml T-DDTs- 0–2.214 ng/ml T-ensodulfans- 0–1.331 ng/ml Methyl parathion- 0–0.513 ng/ml Monocrotophos- 0–0.052 ng/ml Phorate- 0–0.041 ng/ml Butachlor- 0–0.052 ng/ml Chlorpyrifos- 0–0.820 ng/ml Methyl parathion- 0–0.733 ng/ml Phorate- 0–0.646 ng/ml Atrazine- 0–0.160 ng/ml Butachlor- 0–0.077 ng/ml Metalaxyl Fungicide- 0–0.083 ng/ml Chlorpyrifos- 0–0.247 ng/ml Butachlor- 0–0.060 ng/ml	GC-MS/MS	Mondal et al., [88]
Tamil Nadu (Thamirabarani river) Sites: Manimuthar, Tirunelveli, Srivaikuntam, Authoor, and Punnakayal	Surface water	ΣBHC- 0.002–0.059 μg/l ΣHeptachlor- 0–0.08 μg/l ΣEndosulfan- 0.59–34.447 μg/l ΣDDT- 0.006–0.1195 μg/l ΣAldrin- 0.024–2.378 μg/l ΣEndrin- 0.1545–0.317 μg/l ΣCypermethrin- 0.012–0.027 μg/l	GC-MS	Arisekar et al., [9]
Haryana (districts: Hisar, Rohtak, Jind, Mahendergarh, Ambala, and Sirsa).	Water (pond)	Monocrotophos- 0.032–6.38 ppb Pirimiphos Methyl- 0.015–0.121 ppb Fenitrothion- 0.134 ppb Malathion- 0.022–0.119 ppb Chlorpyrifos- 0.013–0.169 ppb Quinalphos- 0.187 ppb Edifenphos- 0.285 ppb	GC-ECD and GC-NPD	Mishra et al., [85]
Gujarat (Tapi river)	Surface water	Endosulfan- 37.56 μg/l Chlorpyrifos- 0.86 μg/l Methyl parathion- 0.43 μg/l	GC-FID	Hashmi et al., [60]
South-West coast (Kerala: Kasargod, Kozhikode, Ernakulam, Kollam, Trivandrum district Tamil Nadu: KanyaKumari district along the agricultural, backwaters and coastal transects)	Soil	ΣHCH- ND−123 ng/g ΣDDT- ND−148 ng/g ΣEndosulfan- ND−21 ng/g Aldrin- ND−108 ng/g Dieldrin- ND−76 ng/g Endrin- ND−113 ng/g Endrin Ketone- ND−143 ng/g Methoxychlor- ND−16 ng/g Heptachlor- ND−139 ng/g Heptachlor epoxide- ND−103 ng/g	GC-MSD	Khuman et al., [73]
Uttar Pradesh (Bisrakh block- Gautam Budhu Nagar, Loni block- Ghaziabad district, Khekhra block- Baghpat district)	Groundwater	ΣHCH- 0.00015–0.000947 μg/l ΣDDT- 0.0013–0.17626 μg/l Chlorpyrifos- 0.00166–0.00537 μg/l Aldrin- 0.00013–0.00042 μg/l	GC-ECD	Srivastava [133]
Punjab (District: Fatehgarh sahib)	Wastewater Soil	Triazophos- 0.0084 ppm Simazine- 0.1612 ppm Fipronil- 0.0338 ppm Atrazine- 0.0045 ppm Carbendazim- 0.0076 ppm Cartap- 0.0058 ppm Chlorpyrifos- 0.0065 ppm Paraquat- 0.0083 ppm Triazophos- 0.0089–0.0128 ppm Simazine- 0.1755–0.2212 ppm Fipronil- 0.0529–0.0686 ppm Atrazine- 0.0044–0.0071 ppm Carbendazim- 0.0089–0.0090 ppm Cartap- 0.0071–0.0072 ppm Chlorpyrifos- 0.0084–0.0089 ppm Paraquat- 0.0079–0.0127 ppm	HPLC	Pahal et al., [99]
West Bengal (Districts- North 24 Parganas and Nadia) New alluvial zone, Eastern India.	River water Pond water	T-HCH−1.26 ng/ml T-DDT- 1.11 ng/ml T-Endosulfan- 0.59 ng/ml Phorate- 0.005 ng/ml Parathion-methyl- 0.031 ng/ml Malathion- 0.015 ng/ml Chlorpyrifos- 0.075 ng/ml Quinalphos- 0.023 ng/ml Profenophos- 0.015 ng/ml Butachlor- 0.025 ng/ml T-HCH- 0.11 ng/ml T-DDT- 0.18 ng/ml T-Endosulfan- 0.03 ng/ml Phorate- 0.005 ng/ml Parathion-methyl- 0.031 ng/ml Chlorpyrifos- 0.13 ng/ml Quinalphos- 0.083 ng/ml Butachlor- 0.047 ng/ml	GC-MS	Bhattacharyya et al., [19]
Rajasthan (Bikaner)	Drinking water	α-HCH- 0.073–0.192 ppm β-HCH- 0.062–0.126 ppm γ-HCH- 0.053–0.139 ppm Dieldrin- 0.023–0.095 ppm Heptachlor- 0.034–0.098 ppm Heptachlor epoxide- 0.044–0.108 ppm Endosulfan- 0.048–0.160 p’p-DDE- 0.063–0.170 ppm p’p-DDD- 0.078–0.159 ppm p’p-DDT- 0.096–0.190 ppm	GLC	Sharma [124]
Uttar Pradesh (Katarniaghat Wildlife Sanctuary)	Water	α-BHC- 0.013–0.203 µg/l δ-BHC- 0.013–0.039 µg/l γ-BHC- 0.014–0.048 µg/l α-endosulfan- ND−0.004 µg/l	GC	Sarkar et al., [122]
Rajasthan (Kota region)	Groundwater	Dimethoate- 0.0103–0.0421 mg/l Chlorpyrifos- 0.0045–0.0175 mg/l Malathion- 0.0032–0.0053 mg/l	GC & HPLC	Sheikh et al., [127]
Uttar pradesh (lucknow, Gomti river)	Surface water	Dichlorvos- 0.0042–0.0067 μg/l Phorate- 0.1121–0.9145 μg/l Chlorpyrifos- 0.0316–0.3864 μg/l Fipronil- 0.0184–0.1616 μg/l Quinalphos- 0.0105–0.1102 µg/l Butachlor- 0.0359–0.1289 μg/l Profenophos- 0.0087–0.1148 μg/l Buprofezin- 0.0172–0.4927 μg/l Ethion- 0.0180–0.0876 μg/l Triazophos- 0.0143–0.1717 μg/l Cyhalothrin- 0.0720–0.1506 μg/l Cypermethrin I- 0.0024–0.1854 μg/l Cypermethrin II- 0.0156–0.1937 μg/l Cypermethrin III- 0.0209–1.8372 μg/l Cypermethrin IV- 0.0173–0.1884 µg/l	GC-TQMS & GC-MS/MS	Gupta et al., [56]
Kashmir Valley (upper Jhelum region)	River water Groundwater Tap water	Dimethoate- 0.019–0.103 μg/l Chlorpyrifos- 0.017–0.185 μg/l Myclobutanil- 0.035–0.420 μg/l Tebuconazole- 0.050–0.396 μg/l Flusilazole- 0.025–0.066 μg/l Difenoconazole- 0.189–0.412 μg/l Hexaconazole- 0.032–0.195 μg/l Spiromesifen- 0.007–0.024 μg/l Fenazaquin- 0.014–0.095 μg/l Quinalphos- 0.011–0.029 μg/l Dimethoate- 0.009–0.010 μg/l Chlorpyrifos- 0.019–0.020 μg/l Myclobutanil- 0.056–0.064 μg/l Tebuconazole- 0.021–0.037 μg/l Flusilazole- 0.014 μg/l Difenoconazole- 0.040–0.051 μg/l Hexaconazole- 0.034–0.038 μg/l Fenazaquin- 0.027–0.028 μg/l Quinalphos- 0.013–0.014 μg/l Dimethoate- 0.005–0.010 μg/l Chlorpyrifos- 0.006–0.008 μg/l Myclobutanil- 0.001–0.012 μg/l Difenoconazole- 0.001 μg/l Hexaconazole- 0.001–0.002 μg/l Fenazaquin- 0.010 μg/l	GC-MS/MS	Ganaie et al., [49]

These studies reliably highlight the extensive prevalence of pollution caused by pesticide applications in Indian agroecosystems, raising substantial concerns about the adverse impacts on the environment and human health. The indiscriminate and excessive use of pesticides, specifically for getting higher crop yields by combating pests and diseases, has ultimately led to soil and water resource contamination across various agricultural regions of the country. Due to their high accumulation potential in soil, the detrimental effects are on soil fertility, microbial activity, and biodiversity. The result is depicted by lower crop productivity and disrupted ecosystem functioning. The contamination of surface and groundwater resources through leaching and runoff of pesticides from agricultural fields, along with defective disposal practices, poses a potential risk not only to aquatic ecosystems but also puts pressure on the availability of safe drinking water sources.

Many studies have also reported the presence of pesticide residues in food products such as food grains, fruits, and vegetables, raising concerns about potential health risks associated with dietary exposure [12], [123], [126], [130], [132], [17], [36]. Further, these residues of pesticides are also detrimental to non-target organisms, such as pollinators, beneficial insects, and wildlife.

## 7 Health effects of pesticide

**Fig. 5:**
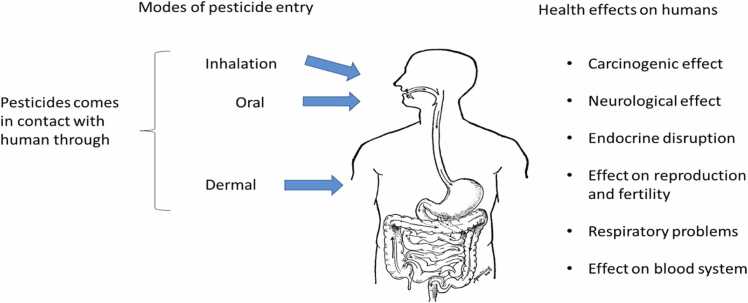
Modes of pesticide entry and health effects of pesticide exposure on humans.

Exposure of humans to agrochemicals causes serious health risks. Humans get exposed to pesticides and other agrochemicals through oral, dermal, and inhalation exposure leading to various health risks (Fig. 5). A meta-analysis study by Xie et al. [157] concluded that pesticide exposure increased the risks of multiple cancers such as prostate cancer and non-Hodgkin lymphoma. Rodrigues et al. [113] reported neurological effects of pesticides such as elevated risk of Parkinson’s disease in Nature Reviews Neurology. Reduced fertility and birth defects were also the side effects of pesticide exposure studied by Jain et al. [64]. Approximately 90 % of the pesticide exposure accounted for dermal exposures [114]. Exposure to pesticides causes many toxicities ranging from mild symptoms (irritation to the skin or allergic reaction) to serious symptoms (strong headache, nausea, dizziness, etc.) and immunological effects increasing susceptibility to autoimmune diseases and infections [47]. Even long exposure to pesticides causes chronic abnormalities in humans, spanning from the development of cancer to various other serious diseases [6].

Pesticide residues show carcinogenic effects and it can develop many kinds of cancer in humans. When pregnant women come in contact with these residues of pesticides there is a potential risk that their child could develop increased sensitivity to conditions like leukaemia, Wilms' tumour, and brain cancer. The primary link between external pesticide exposures and childhood blood cancer has been well established [134]. Weichenthal et al., [153] found out that 19 out of 32 pesticides have been linked to the occurrence of at least one cancer, encompassing bladder, colorectal, lung, brain, melanoma, pancreatic cancer, leukaemia, multiple myeloma, and non-Hodgkin lymphoma.

Various studies have been conducted and revealed that areas with heavy agrochemical use had the highest cancer rates in contrast to regions where the use of such cancer-causing compounds was minimized. This research further reinforced and substantiated the evidence linking pesticides to the cancer risk [100]. A notable research study demonstrated a correlation between a higher risk of childhood cancer on exposure to pesticides [134]. Mostly all organochlorine pesticides have a fat-soluble (lipophilic) nature and they remain in the adipose tissue for decades due to their bioaccumulation the residues of OC from the adipose tissue reach to bloodstream and might mix with the breast milk and can be injurious to the infant [91].

Common and typical symptoms have been shown by the pesticide residues. Direct exposure to pesticides may cause symptoms such as irritation and reddening of the eye, headache, hazy vision, and contraction of the pupil. The common symptoms caused by general poisoning appear due to the accumulation of neurotransmitters at nerve endings. Severe poisoning, however, may lead to pale skin, increased sweating, and a foaming mouth. Other symptoms include alterations in vital signs, such as weakness in muscles, seizures, confusion, and loss of consciousness. Without prompt treatment, the victim may face a fatal outcome [6].

The OPs and dithiocarbamate interrupt peripheral nerve conduction and a few related disorders [159]. A survey study conducted by Buralli et al., [25] on the Brazilian population strongly determined that mental disorders are linked to the presence of pesticides in the blood cholinesterase of each surveyed individual.

Pesticide residues also disrupt the endocrine functioning. Endocrine hormone is essential for our body and is involved in many bodily processes particularly those related to the reproductive and growth function. There were substantial indications of DDT and its isomers like DDE being implicated in the disturbance of endocrine glands [142]. Almost all classes of OCs found were discovered to be linked to the abnormal operation of the endocrine system, even at very low residual levels [76]. Additionally, Endocrine gland disruption and malfunctioning have also been linked to organophosphates such as parathion and malathion [51]. A study conducted by Slotkin and Seidler [131] revealed that the presence of organochlorines e.g., dieldrin at micromolar levels causes a significant decrease in cell growth and function. Another study inferred that heptachlor and dieldrin were responsible for inducing increased apoptosis in cell cultures, leading to oxidative stress and damage to mitochondria [35].

Exposure to pesticides is also reported to affect reproduction and fertility. These chemicals are found to be associated with changes in fetal growth, birth imperfections, and craniate death. A herbicide mixture of 2,4,5-trichlorophenoxyacetic acid and 2,4-dichlorophenoxyacetic acid in a ratio of 50:50 was observed to be associated with severe health and genetic consequences in a study conducted in Vietnam and Malaya. Also, the offspring who were vulnerable and came in contact with pesticides showed defects in biological processes and birth weight (pesticides [134]. Exposure to these toxic chemicals also causes a reduction in male fertility, a decrease in the variety of spermatozoon, germinal animal tissue injuries, and disrupted endocrine functioning. Long-term exposure to these pesticides is responsible for being the causative factor of Alzheimer's and Parkinson's diseases [116].

The well-being of agricultural communities, consumers of farm products, and the general population is seriously threatened by the health implications caused by exposure to pesticides and their residues. It, therefore, emphasizes the urgent need for holistic and integrated initiatives to reduce pesticide contaminations.

## 8 Impacts of pesticide residues on biodiversity

As the residues of pesticides are stable in the environment, they often remain persistent in behavior and show dangerous effects on non-target and non-pest organisms [149]. These chemicals are highly toxic to organisms (mammals, amphibians, birds, fish, and insects) on exposure. Bioaccumulation is defined as the accumulation or steady increase of substances, such as pesticides or other chemicals, over time. Accumulation in organisms occurs when the rate of intake of any substance is greater than its rate of elimination by catabolism and excretion More precisely, bioaccumulation is a process whereby a chemical substance is absorbed within an organism through all routes of exposure, including dietary absorption, transport across respiratory surfaces, and dermal absorption. This includes bioconcentration (uptake from water) and biomagnification (uptake through food) [69]. Whereas, biomagnification, also called bioamplification or biological magnification, is the progressive rise in concentration of a substance in living organisms at successively higher trophic levels of a food chain. This results in an accumulation of contaminants within a food chain as a contaminant advances up through many trophic transfers. Consequently, high substance levels will accumulate at higher trophic levels, for instance, top predators, compared with lower trophic levels [55]. It has been recorded that due to pesticide application in Germany, there is a decline of 70 percent in insect biomass whereas in Europe there is a decline of 50 percent in farmland birds [6].

Gibbs et al., [54] studied that from 1980 to 2006 there was an average loss of 10 percent in species of common birds but in 2006 the decrease of farmland bird species was recorded more than 50 percent in the U.K, indicating the adverse impact of pesticides on the environment. Similarly, there was a reduction in regional diversity of invertebrates of about 40 % in streams and rivers due to the use of currently used pesticides (CUPs). Similarly, in Europe, exposure to pesticides, even at environmentally safe levels, resulted in a 42 % reduction in species richness [15]. It was estimated that approximately 72 million birds in the United States lost their lives annually due to pesticide application [48].

DDT and dieldrin (insecticides) usage resulted in the decline of many bird species such as Falco peregrinus and fish-eating birds in some regions of America and Europe [95]. Liquid form of carbofuran showed fatal effects on burrowing owls [53]. The adverse effect of diazinon application on grasslands resulted in the highest mortality among the Brant geese population, which previously nested in that area to lay their eggs [137].

The tissues of prey may accumulate pesticides, which can have deleterious consequences on top predators. Rodenticide pesticides in particular are extremely poisonous and accumulate within the bodies of rodents. These chemicals may lead to secondary poisoning of predators such as foxes, dogs, raptors, and unintended mammals when they consume prey that has been exposed to pesticides [21]. The life of common shrews, badgers, and wood mice got impacted because herbicides damage the plants which reduce food availability and alters the microclimate [62].

In China, the HCH and DDT residues were detected in four distinct fish species inhabiting freshwater regions. HCH residue content was detected higher in club white and grass carp as compared to DDT residues which was found to be greater in fish (snakehead species) [156]. Furthermore, between 1994 and 2004 in Brazil, DDT, HCH, and chlordane residues were detected in the blubber of franciscana dolphins [77].

One-third of Amphibian species are endangered because of many reasons such as the destruction of habitat, over-exploitation, and introduction of predator species by pesticide usage. The important element in this situation is water pollution resulting from the runoff and seepage of pesticide residues [24]. Besides the insecticides, fungicides such as difenoconazole and herbicides such as diclofop-methyl also demonstrated adverse effects on the albino rats. The examined rats’ metabolic and enzymatic functions were interfered with by these substances. There is a significant potential for these substances to induce toxicity in both humans and the environment [1].

The aerial use of certain pesticides led to the complete elimination of arthropods in various crops, including cotton. In predator species such as *Chrysoperla carnea*, secondary poisoning occurs resulting from systemic insecticides, which consumed insects exposed to pesticides and faced threats from agrochemicals in agricultural ecosystems [80]. Various findings of research revealed that insecticides such as cartap, flubendiamide, abamectin, and imidacloprid were harmless while rynaxypyr and pyriproxyfen were classified as non-injurious. Pymetrozine was rated as having a moderate effect on bugs [89].

It has been extensively documented that besides a reduction in cereal yield due to 50 % decrease in plant populations, the number of insects was reduced by 33 %, and that of species of birds was reduced by approximately 20 % in the United Kingdom [112].

Pesticides have severe consequences on honey bees. The larvae and adults of Wild honey (*Apis dorsata*) exhibit metabolic alteration due to the influence of diafenthiuron, imidacloprid, and etofenprox. It was observed that bee hemocytes were notably impacted as shown by weakened immune systems against diseases and various abnormalities, comprising distorted cell shapes, agglutination, and denucleation [104].

The influence of neonicotinoids on *Apis mellifera* was studied, and it was found that imidacloprid and thiacloprid decrease the count of antimicrobial effectiveness, hemocytes, and encapsulation response. These abnormalities, particularly at greater concentrations, were attributed to the impact of Clothianidin [22]. Neonicotinoids were linked to disturbances in multiple sensory functions and showed an adverse impact on the feeding behaviour of pollinators [38].

It can be inferred from the studies that pesticides cause detrimental effects on human health and also lead to biodiversity loss.

Various studies have been conducted in India to analyze the presence of different pesticide residues in water and fish. A few examples are: [9] studied the accumulation of residues of OC pesticides in the water, sediments, and fish of the Thamirabarani river system of southern peninsular India. The study revealed that the most abundant OCPs in the surface water and sediments are Aldrin, Endosulfan, heptachlor, endrin, and dieldrin. The major OCPs that showed in both are endosulfan. Also, the bioaccumulation of endosulfan and endrin was found to be higher in fish tissues (catla, rohu, and tilapia). A general pattern for bioaccumulation of OCPs was seen in freshwater fishes showing the following order: rohu > tilapia> catla.

The study of Gomti River in UP by [94] detected the presence of OC and OP residues such as HCH isomers, DDT and its metabolites (DDD & DDE), endosulfan, chlorpyrifos, and methyl parathion in the Water samples. Also, they recorded the presence of HCH isomers, DDT and its metabolites, endosulfan isomers and metabolite from the OCs, and chlorpyrifos, malathion, dichlorvos, methyl parathion, dimethoate, quinalphos from the OPs in the seven groups of fishes (catfishes, carps, minnows, barbs, spiny eels, gobies, and cichlids).

## 9 Monitoring and management of pesticides

A comprehensive and stringent approach is the need of the hour to address the issues and consequences associated with pesticide pollution in Indian agroecosystems. Effective monitoring techniques including advanced analytical methods, such as gas chromatography-mass spectrometry (GC-MS) and high-performance liquid chromatography (HPLC) are crucial for accurate identification and measurement of even trace amounts of pesticides and their residues in environmental components (soil, water, and agricultural products). The management of pesticide pollution can be effectively implemented by adopting integrated pest management (IPM) practices. IPM is widely acknowledged as a sustainable approach to pest control since it combines chemical, biological, and cultural control with the prudent use of pesticides as a last resort. This strategy encourages the use of environmentally acceptable substitutes including biopesticides, pheromones, and biological control agents while simultaneously reducing reliance on chemical pesticides.

Phytoremediation and bioremediation techniques can also play a vital role in mitigating pesticide pollution. These techniques can effectively and efficiently utilize the potential of suitable plants and associated microorganisms respectively to eliminate or degrade pesticides from contaminated soil and water.

Further improvement in pest management techniques in Indian agriculture can be attained by developing and using biodegradable and environmentally friendly pesticides as well as the investigation of sophisticated formulations with lower environmental impact.

The European Union and the United States have taken diverse programs into action that address the problems with the use of pesticides. The Approach of European Union to tackle this problem are: Legal Framework: The EU approach mainly depends on Regulation (EC) No 1107/2009 relating to the placing of plant protection products on the market and Directive 2009/128/EC establishing a framework for Community action to achieve the sustainable use of pesticides [44], [45]. Hazard-Based Approach: The EU has adopted a hazard-based approach, where substances are banned based on their intrinsic hazardous properties without an investigation of the exposure levels based on intrinsic hazardous properties [59]. Integrated Pest Management (IPM): The EU demands IPM principles that apply pesticides as the last resort, in addition to alternatives that are non-chemical in nature [13]. Reduction Targets: The EU's Farm-to-Fork Strategy aims at reducing the overall use and risk of chemical pesticides by half (50 %) and also those of a more hazardous nature by half (50 %) by 2030 [42]. Promoting Organic Farming: The EU has targeted farmland for up to 25 % of total farmland in organic farming by 2030 [42]. Strict MRLs: The EU ensures strict Maximum Residue Levels in food as well as feed on the use of pesticides [43]. Whereas the approach adopted by the US to combat the problem of pesticides is: Regulatory Framework: The primary regulatory mechanisms for pesticides in the US are the Federal Insecticide, Fungicide, and Rodenticide Act (FIFRA) and the Federal Food, Drug, and Cosmetic Act (FFDCA) [145]. Risk-Based Approach: Under the risk-based approach in the US, pesticide assessments are linked to both hazard and exposure [97]. Registration and Re-evaluation: The EPA registers pesticides based on scientific evaluations and periodically re-evaluates them [146]. Integrated Pest Management: Although not mandatory, the US promotes IPM through various programs and projects [144]. Residue Monitoring: FDA constantly monitors the residues of pesticides in food supply [148]. Worker Protection: Occupational Standards for Workers in Agriculture and Pesticide Handlers. The Protection Standard for Agricultural Workers and Pesticide Handlers was first launched by US EPA [147].

## 10 Regulatory and policy framework

There is an urgent need for holistic policy reforms in India on pesticide use, making productive agriculture congruent with environmental as well as health concerns. Major changes include sterner enforcement of bans already enacted on hazardous pesticides through task forces, traceability systems, and increased penalties. The process of registering pesticides shall be strict, and more transparent, with periodic reviews, and a thorough environmental assessment.

The Insecticides Act of 1968 and the Insecticides Rules of 1971 govern India's regulatory framework for pesticides. The Central Insecticides Board and Registration Committee (CIBRC) is responsible to undertake and monitor the registration and regulation process of pesticides. The regulatory frameworks and enforcement mechanisms need to be strengthened to manage and moderate the use of pesticides. This includes enforcing stringent regulations for the use, storage, and disposal of pesticides, conducting periodic evaluations of toxicity data and environmental impacts, updating the processes and procedures for pesticide registration, and encouraging programs to raise awareness and educate farmers and other stakeholders.

Policy recommendations are required to emphasize the promotion of sustainable agriculture practices through: Stronger incentives for organic farming; financial support; national organic farming policy; and crop insurance schemes especially tailored to specialized crops. IPM should be widely promoted through: Mandatory training; increased research funding; and a national certification program. These can be strengthened by providing incentives and support for the adoption of eco-friendly alternatives to chemical pesticides. Increasing the number of testing laboratories, having a national database, and aligned maximum residue limits can enhance pesticide residue monitoring. So, it's important to give financial support to research and development of safer, biodegradable pesticide formulations. In addition, education among farmers should improve through better digital platforms, the extension services should be strengthened, and anyone engaged in agriculture should be trained mandatorily. That is why prioritizing broad education and awareness programs to emphasize the dangers of pesticide misuse and support sustainable farming methods can also prove to be supportive. Worker protection can be enhanced by implementing strict safety standards and protection equipment, which they have to wear. More registration and research should be invested in biopesticides and safer alternatives. It is highly essential to promote cooperation on an international level, harmonize pesticide laws with surrounding nations, and uphold international agreements such as the Rotterdam and Stockholm Conventions. The policies related to pesticide manufacturing, storage, and usage must be reviewed and updated regularly. They should revolve around the present status of changes in agriculture to forecast the fate, transport, and potential effects of pesticides in various environmental compartments, and to enable proactive mitigation efforts, modelling, and risk assessment studies are essential. These policy improvements would save the environment and minimize the adverse effects of pesticide use if implemented correctly. They are meant to bring numerous positive impacts on agricultural productivity and, therefore, require the input of all stakeholders. Therefore, the implementation should be gradual and continuously monitored and evaluated to make adjustments on effectiveness.

## 11 Future research directions

Pesticide contamination in Indian agroecosystems can be addressed through a multifaceted approach integrating research across various disciplines. Development and implementation of state-of-the-art technologies such as high-resolution mass spectrometry and biosensors can help identify and measure trace levels of pesticides, their residues, metabolites, and the transformation products with high accuracy in environmental matrices (soil, water, and agricultural products).

Exploration of natural sources such as plant-derived compounds and microbial metabolites to develop biodegradable pesticide formulations with minimal environmental persistence and toxicity can be thoroughly researched. Also, modelling, risk assessment, and impact assessment studies are essential to predict the fate, transport, and potential effects of pesticides in various environmental compartments and to strategize proactive mitigation efforts.

Investigating the role of plant-associated microorganisms in improving and enhancing the efficiency of phytoremediation and biodegradation techniques for removal or degradation of pesticide contaminants is required to provide insights into sustainable remediation. Research and innovation are also required in the areas of molecular mechanisms focussing on pesticide toxicity and biomarkers development for early detection of exposure and adverse effects. This can be a prominent strategy to safeguard human and environmental health.

Collaboration among multidisciplinary subject experts encompassing fields such as chemistry, biology, environmental science, and agriculture, will be fundamental in addressing the multifaceted challenges posed by pesticide contamination in Indian agroecosystems to pave the way for sustainable and resilient agricultural practices.

## 12 Conclusion

Pesticides, while convenient for pest management in agriculture, have significant environmental implications as only 0.1 % reaches target organisms while the remainder disperses into ecosystems. This decade-spanning review reveals widespread contamination across Indian states, with OCPs and OPPs being predominant pollutants due to their persistence and bioaccumulative nature. The study documented varying levels of pesticide residues, including carbamates and synthetic pyrethroids, in groundwater, surface water, and soil across different states, indicating potential environmental and human health risks. To address these challenges, several key interventions are recommended: implementing bioremediation and phytoremediation technologies, enhancing farmer education on proper pesticide usage, enforcing strict measures against banned pesticide sales, promoting Integrated Pest Management practices, and maintaining regular monitoring of soil and water quality. The compiled data serves as a valuable resource for future research and policy interventions aimed at mitigating pesticide pollution in Indian agroecosystems.

The findings emphasize the urgent need for better control measures and sustainable agricultural practices. Continuous monitoring and analysis of soil and water quality, combined with improved awareness and training programs, are essential for reducing pesticide residues in the environment and protecting both ecosystem and human health.

## CRediT authorship contribution statement

**Pooja Arora:** Writing – review & editing, Supervision, Conceptualization. **Urvashi Kashyap:** Writing – original draft. **Shivani Garg:** Writing – review & editing, Validation.

## Declaration of Competing Interest

The authors declare that they have no known competing financial interests or personal relationships that could have appeared to influence the work reported in this paper.

## Data Availability

Data will be made available on request.
